# Tumor driven by gain-of-function HER2 H878Y mutant is highly sensitive to HER2 inhibitor

**DOI:** 10.18632/oncotarget.5221

**Published:** 2015-09-08

**Authors:** Zexi Hu, Yong Hu, Xicheng Liu, Rongwen Xi, Aiqun Zhang, Deruo Liu, Qiang Xie, Liang Chen

**Affiliations:** ^1^ College of Life Sciences, Beijing Normal University, Beijing, China; ^2^ National Institute of Biological Sciences, Beijing, Beijing, China; ^3^ The General Hospital of People's Liberation Army (301 hospital), Beijing, China; ^4^ Department of thoracic surgery, China-Japan Friendship Hospital, Beijing, China; ^5^ Fuzhou Pulmonary Hospital of Fujian, Fujian, China; ^6^ National Institute of Biological Sciences, Collaborative Innovation Center for Cancer Medicine, Beijing, China

**Keywords:** HER2, H878Y, Transgenic mouse model, targeting therapy, combinational therapy

## Abstract

HER2, a well established oncogenic member of EGFR family, is among the most intensely investigated kinase drug targets. In contrast to hotspot mutations of EGFR, few mutations of HER2 locate in activation loop within kinase domain. We previously reported the molecular mechanism underlying hyper kinase activity of HER2^H878Y^, a mutation located in activation loop. However, its tumorigenicity *in vivo* and relevant therapeutics remain to be determined. Here, we report for the first time that HER2^H878Y^ was tumorigenic *in vivo* in lung adenocarcinoma transgenic mouse model. Induced expression of HER2^H878Y^ in lung epithelial compartments resulted in formation of poorly differentiated lung adenocarcinoma with bronchioloalveolar carcinoma (BAC) features. Strikingly, we found that these tumors depended on continuous expression of HER2^H878Y^ for maintenance. Typical HER2 downstream signaling mediators, including PLCγ1, STAT5 and AKT, were hyperactivated in HER2^H878Y^ driven lung tumors. More importantly, administration of HKI-272, a tyrosine kinase inhibitor (TKI), efficiently shrank HER2^H878Y^ driven tumors in transgenic mouse model. Moreover, we found that combinational treatment with HKI272 and mTOR inhibitor, Rapamycin, showed a superior cytotoxicity to H878Y mutant transformed cells and enhanced activity to elicit apoptosis and inhibit growth *in situ* in tumorous area. Our work therefore showed that HER2^H878Y^ mutant was a reasonable drug target. Hence, our work supported the assessment of HKI-272/rapamycin treatment in clinical trials.

## INTRODUCTION

HER2, a 185-Kd transmembrane receptor tyrosine kinase of the ErbB family, is a heterodimer partner to other ErbB family members to prolong and enhance downstream signaling with ampliative ligand specificity [1], resulting in activation of several downstream signaling pathways, including MAPK, PKC and AKT pathways. HER2 thus plays important roles in cell division, migration and differentiation at both cellular and organismal level [1, 2].

Numerous studies reported that HER2 was amplified and overexpressed in several types of cancer, especially in breast cancer [2-4]. Consistently, overexpression of HER2 could continuously activate cellular proliferation and survival pathways, resulting in cell transformation and evasion from cell death [5]. Interestingly, HER2 was recently reported to be overexpressed in lung cancers with acquired resistance to EGFR targeted therapy that lack the secondary T790M mutation [6].

In addition to overexpressed status, mutations were also found to activate HER2 signaling loop and drive tumorigenesis. Bose et al. found seven HER2 somatic mutations in breast cancer lacking *HER2* gene amplification [7]. These mutations strongly increased phosphorylation of HER2 downstream signaling proteins, including PLCγ and MAPK, indicating that these are activating mutations; all of these mutations are sensitive to HER2 inhibitor HKI-272 (neratinib), including the lapatinib resistant mutation HER2 L755S [7]. Moreover, HER2 mutation (mainly G776insYVMA mutation) was found in 2∼4% lung cancer patients [8-10]. Most of these reported mutations were located in HER2 extracellular domain and kinase domain, but not in the activation loop [7], which is in contrast to several prominent oncogenic mutations, such as BRAF V600E and ALK R1275Q,moreover,the hotspot mutation, L858R [11] in EGFR locates in its activation loop.

Recently, H878Y mutation in HER2 was reported in 11% of hepatocellular carcinoma patients [12]. The mutation results in the mutant HER2 to harbor Y877/Y878 motif in activation loop, similar to wild-type Y1007/Y1008 in the JAK2 kinase [13]. Our previous work have shown phospho-Y878 forms a salt bridge with the adjacent R898 residue to stabilize the kinase in a permissive conformation, thus conferring an enhanced kinase activity for HER2 [14].

Despite of the previous biochemical characterization, whether H878Y mutant HER2 is tumorigenic in mouse level and relevant therapeutics remain to be determined. HER2 transgenic mouse models reported in earlier studies mainly focused on wild-type HER2 in breast cancer [2] and the recent HER2 G776insYVMA mutation in lung cancer model [10]. Here we reported a doxycycline inducible H878Y transgenic mouse model. We showed that overexpression of H878Y mutant HER2 resulted in formation of poorly differentiated lung adenocarcinoma with bronchioloalveolar carcinoma (BAC) features and that tumors were dependent on continuous expression of mutant HER2 for maintenance. We further showed that tumors driven by HER2 H878Y mutant were sensitive to HKI-272. We also showed that combinational treatment with HKI-272 and Rapamycin resulted in enhanced toxicity to HER2 H878Y *in vitro* transformed cell lines and *in vivo* tumors.

## RESULTS

### HER2^H878Y^ is sensitive to HER2 inhibitors

HER2 is a more potent oncogene than other ErbB family members [1], and amplification of HER2 was reported in several types of cancer. Overexpression of HER2 transforms normal mammary epithelial cell and induce breast cancer in mouse model [2]. Earlier reports by us and others showed that HER2^H878Y^(H878Y hereafter) is transforming *in vitro* [14, 15]. We established stable cell lines to overexpress wild-type and H878Y mutant HER2 in two normal cell lines (NIH-3T3 and BEAS-2B) and checked their ability to form colonies in soft agar system. Consistent with our earlier report, we found that H878Y was more potent than wild-type HER2 to transform both of the cells (Supplementary Figure. 1A and 1B). In addition, these stable H878Y over-expressed cell lines showed markedly enhanced downstream signals, including phosphorylated PLCγ and STAT5 (Supplementary Figure. 1C). These data again confirms H878Y is a gain-of-function mutation.

**Figure 1:**
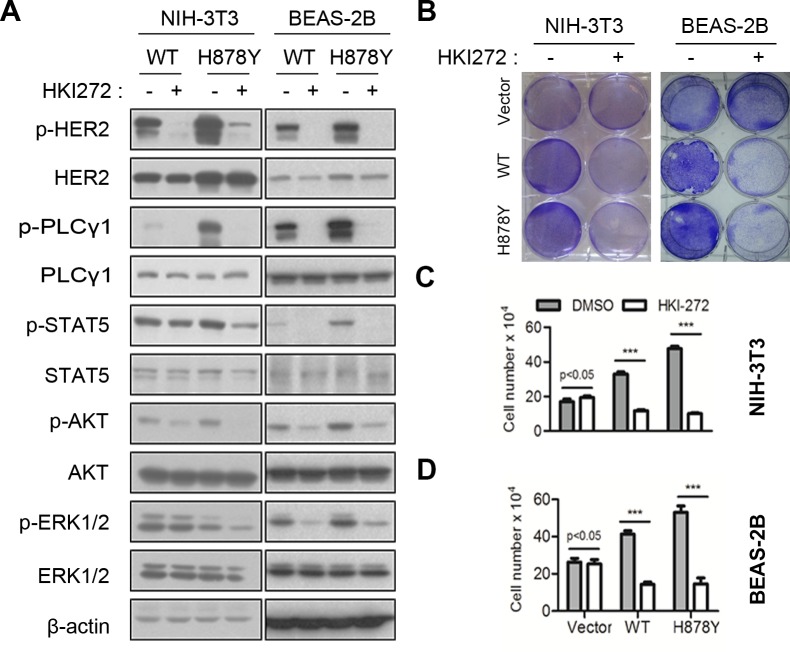
HER2 H878Y is sensitive to HKI-272 **A.** HER2 stable cell lines were treated with 500nM HKI-272 for 30 minutes, and then subjected to immunoblotting for detecting HER2 downstream signalings. **B.**, **C.** and **D.** HER2 stable cell lines were treated with 500nM HKI-272 for 3 days, cells were fixed with 4% PFA and then stained with 0.01% crystal violet **B.**, or digested to count cell numbers **C.** and **D.**. ***P < 0.001.

While wildtype HER2 overexpressed tumor cells were sensitive to HER2 inhibitors, those transformed cells by mutants like V777L, G776insYVMA and truncated isoform were less sensitive or resistant to HER2 inhibitor lapatinib [9, 16, 17]. In order to determine the sensitivity of H878Y to HER2 kinase inhibitors, we treated HER2 expressed cell lines with HKI-272 (Neratinib), an irreversible dual inhibitor of HER2 and EGFR currently tested in clinical trial. Consistent with our earlier report, short-term treatment (30 min) efficiently inhibited HER2 elicited signals (Figure 1A), suggesting that both wild-type and H878Y HER2 were sensitive to HKI-272 inhibition. Consequently, we observed reduced phosphorylation of AKT and ERK1/2, two canonical proliferation signals downstream of HER2. Meanwhile, a long-term (3 days) HKI-272 treatment dramatically inhibited proliferation of transformed cells (Figure 1B and 1C). Our data therefore demonstrated that H878Y mutation conferred higher oncogenic activity and elicited stronger downstream signals in comparison with wild-type HER2. More importantly, H878Y mutation was also sensitive to HKI-272 *in vitro* cells.

### HER2^H878Y^ drives formation of lung adenocarcinoma in transgenic mouse model

Given that HER2 H878Y is a gain-of-function mutation and H878Y mutation is also found in a portion of cancer patients, it would be interested to test whether H878Y is tumorigenic *in vivo*. Previous research had reported that HER2^YVMA^ mutant could drive rapid development of lung adenosquamous with intrabronchial carcinomas in the proximal and distal brochioloalveolar locations [10]. The notable difference of mutation spot within HER2 kinase between HER2^YVMA^ and H878Y (HER2^YVMA^ in kinase domain versus H878Y in activation loop) motivated us to test tumorigenecity of H878Y mutant HER2 *in vivo* with transgenic mice.

**Figure 2:**
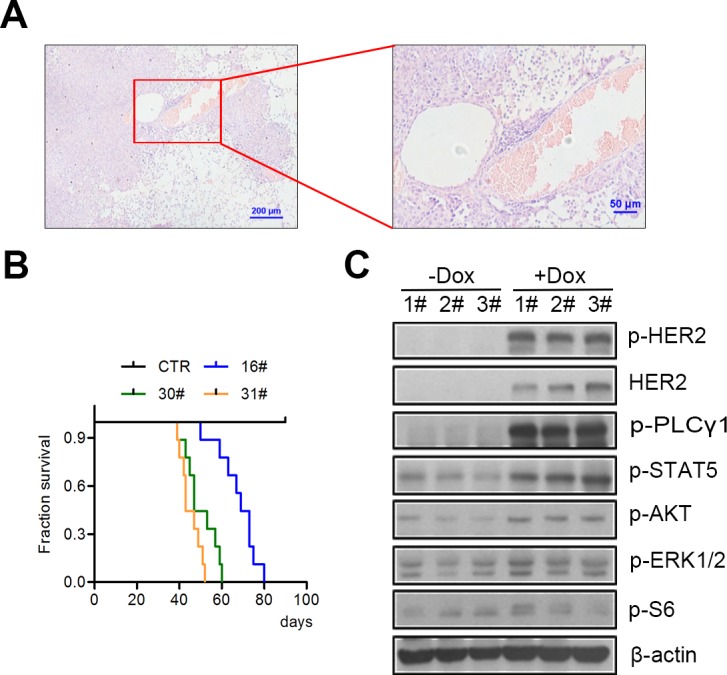
H878Y mutant HER2 is tumorigenic *in vivo* **A.** A representative H&E image of lung tumor of H878Y transgenic mice. Scale bar: 200μm for left panel, 50 μm for right panel. **B.** Kaplan-Meier survival curves for three different H878Y transgenic mouse founders. 16#, 30# and 31# had median survival latency of 69, 47 and 43 days respectively. *n* = 9 mouse for each founder line. **C.** H878Y bitransgenic mice were treated with doxycycline-containing or normal food for 4weeks, and then lung tissues were collected for immunoblotting of HER2 related signaling. Samples were from three different offspring of 31# H878Y founder line.

We therefore utilized the inducible ‘tet-on’ expression system to generate *tet-op hHER2^H878Y^* transgenic mice, in a similar way with the reported HER2^YVMA^ transgenic mice. To achieve this goal, an open reading frame (ORF) encoding human H878Y HER2 was cloned in between a TetO promoter/β-globin intron and a SV40 polyA cassette (Supplementary Figure. 2A). The linear DNA fragment was microinjected into FVB/N fertilized eggs to obtain transgenic mouse founders (Supplementary Figure 2B). The resulting *tet-op hHER2^H878Y^* founders were then crossed with CC10-rtTA mouse to generate bitransgenic mouse for specific overexpression of H878Y transgene within lung epithelium in a doxycycline-inducible manner [18]. We had successfully applied this system in several of our studies and found that this system was capable of tight control of the expression of the transgene [19, 20]. Moreover, targeted therapy developed based on this system was widely applicable to various types of tumors, but not limited to lung cancer study [21, 22]. Among 10 available transgenic mouse founder lines, *CC10-rtTA/tet-op hHER2^H878Y^* pups of three founder lines efficiently developed lung tumors when fed on doxycycline (Dox hereafter)-containing diet for about 10 days. All of those mouse eventually died after Dox induction for around 2.5 months. After Dox treatment for one month, we sacrificed the mouse to examine the pathology of the lungs. We detected poorly differentiated lung adenocarcinomas with features of diffused bronchioloalveolar carcinoma in H878Y transgenic mouse (Supplementary Figure 2C), which was in strikingly contrast to those seen in HER2^YVMA^ transgenic mice (glandular and squamous differentiation pathology with *in situ* tumor mainly located in proximal and distal airway epithelia [10]). These results were also confirmed with thorough analysis of the pathology of lungs from mouse fed on Dox for around 2 months (Figure. 2A), suggesting different mechanisms underline tumorigenesis driven by H878Y and YVMA mutant HER2 *in vivo*. In addition, bitransgenic pups of all three H878Y founder lines had a medium survival below 10 weeks after Dox induction (Figure. 2B). Additionally, Immunoblotting of lung tissue lysates from tumor bearing transgenic mouse had been detected with activated HER2 downstream signals (Figure 2C), consistent with the result from *in vitro* cell lines.

### H878Y driven lung tumors are dependent on continuous expression of transgene for maintenance

**Figure 3:**
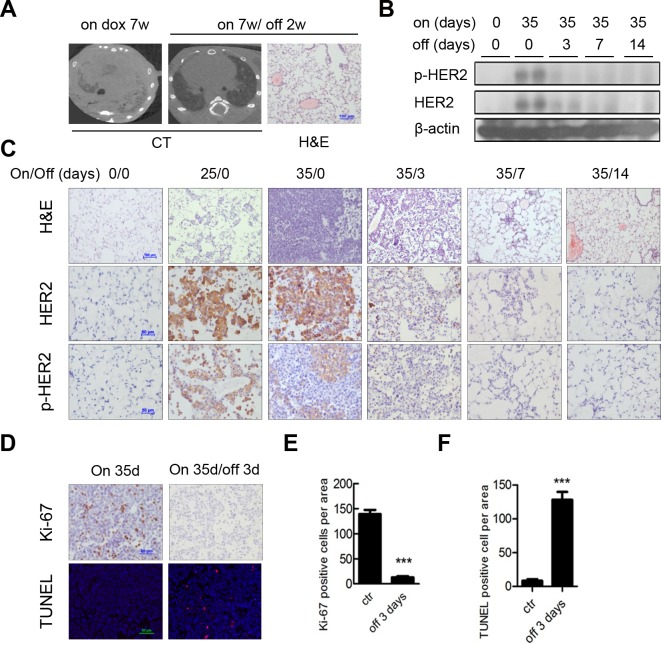
HER2 H878Y driven lung tumor depends on continuous expression of transgene for maintenance **A.** Doxycycline withdrawal for 14 days resulted in a complete tumor regression in HER2 H878Y mice. Images showed CT scanning of one representative mouse that continuously received 7 weeks of doxycycline diet, and then underwent doxycycline withdrawal for another 14 days (left panels). H&E staining showed no evident tumor in the lung of mice with doxycycline withdrawal (right panel). **B.** Immunoblotting analysis of HER2 and phos-HER2 expression in lungs of H878Y bitransgenic mice. Representative data were derived from offspring of 31# H878Y founder line fed on normal food or on doxycycline food for the indicated days. **C.** Kinetics of HER2 and phosphorylated HER2 expression were revealed in *in situ* tumor nodules. Scale bar for H&E was 100μm and IHC was 50μm. **D.** Ki-67 staining (top panel) and TUNEL staining (bottom panel) on H878Y bitransgenic mice on doxycycline food for 35 days and bitransgenic mice on doxycycline food for 35 days and then subjected to doxycycline withdrawal for 3 days revealed dramatically decreased in Ki-67-positive cells and increased in TUNEL-positive cells after doxycycline withdrawal. **E.** Quantification of Ki-67 positive cells. Data of six random tumor-containing fields were counted and analyzed. Values were mean ± SEM (*n* = 6). **P < 0.01. **F.** Quantification of TUNEL positive cells. Data of six random tumor-containing fields were counted and analyzed. Values were mean ± SEM (*n* = 6). **P < 0.01.

Addiction to driver oncogene was reported in various types of cancers. Thus, we assessed whether H878Y induced tumors were dependent on continuous H878Y expression for tumor maintenance. We generated a cohort of bitransgenic mouse and fed them with Dox to induce lung cancer for one month. The tumor burden was documented through CT imaging (Left panel, Figure 3A). Strinkingly, we observed that these lung tumor completely regressed in response to withdrawal of Dox for 2 weeks (middle panel, Figure 3A), indicating that H878Y continuous expression was essential for tumor maintenance. Histology analysis revealed largely normal lung, with occasional areas of thickened alveolar wall and fibrosis (right panel, Figure 3A), indicating remodeling of lung tissues.

Next, we wondered whether discontinuing HER2 expression led to a reduction of HER2 signal. To validate our hypothesis, we analyzed HER2 expression and function in these mouse at different time points. We collected lung tissues from mouse fed on Dox for 35 days, on Dox 35 days/off Dox for 3, 7, or 14 days serial treatment as well as normal diet treatment, and analyzed HER2 and phospho-HER2 expression through Western blotting. We found that HER2 signal arose from undetectable in the mouse fed on normal diet to strong expression level in mouse on continuous Dox for 35 days, and rapidly waned after Dox withdrawal (Figure 3B). To validate these phenomena *in situ* in tumor tissues, we analyzed HER2 and phospho-HER2 levels in lungs through immunohistochemistry (IHC). Lung tissues from bitransgenic mouse without Dox induction were negative of HER2 and phospho-HER2 signals. In contrast, we detected strong HER2 and phospho-HER2 activated signals in mouse fed on Dox for 25 days. After 35 days Dox induction, strong HER2 and phospho-HER2 signals were detected in poorly differentiated lung adenocarcinoma in the bitransgenic mouse. Strikingly, withdrawal of Dox for just 3 days led to regression of HER2 signals to the baseline level, and remained undetectable by 7 and 14 days after Dox withdrawal (Figure 3C).

To determine the biological mechanism underlying tumor regression, we assessed the cellular proliferative/apoptotic index in the tumor regions of mouse after withdrawing Dox. As short as for 3 days of Dox withdrawl, Ki-67, a classical cell proliferation marker, was dramatically downregulated in tumors (Figure 3D, top panel; quantification in Figure 3E). Conversely, the number of apoptotic cells within tumor were increased by TUNEL (terminal deoxynucleotidyl transferase dUTP nick-end labeling) assay (Figure 3D, bottom panel; quantification in Figure 3F).

Taken together, these data showed that H878Y-driven tumors required the continuous function of HER2 mutant for tumor maintenance, suggesting that targeted HER2 H878Y signaling maybe a valid option.

### hHER2^H878Y^-driven tumors are sensitive to HKI-272 treatment *in vivo*

**Figure 4:**
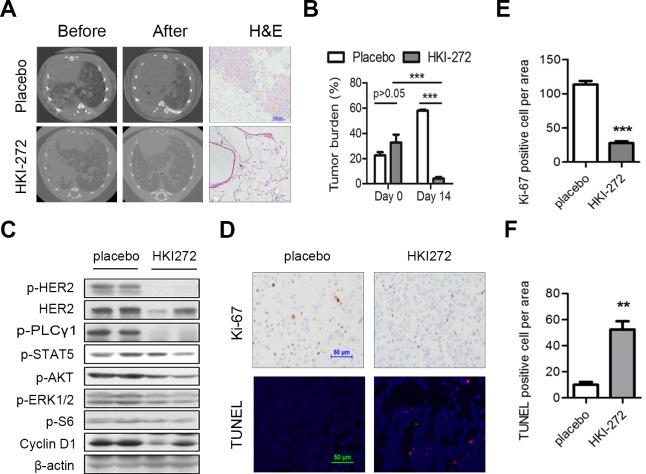
HER2 H878Y-driven lung tumor is sensitive to HKI-272 treatment **A.** HKI-272 treatment led to regression of lung tumor in transgenic mice. Mice were fed with doxycycline food for 4 weeks, and then treated with vehicle or HKI-272 at 50 mg/kg for 2 weeks respectively. Three mouse of each treatment were imaged with CT. Scale bar for H&E picture, 100 μm. **B.** Quantification of tumor areas shown in **A.**. ImageJ software was used to determine tumor areas in the CT images and the percentage of tumor areas against the whole lung was calculated. Values were mean ± SEM (*n* = 3). ***P < 0.001. **C.** Immunoblotting analysis of lung tissues from H878Y-driven tumors. HER2 H878Y mice were treated with vehicle and HKI-272 for three days. Lung tissues were analyzed through western blot. **D.** Representative photographs of cross-sectional tumors assayed for Ki-67 and TUNEL from mouse mentioned in **C.**. Scale bars, 50μm. **E.**, **F.** Quantification of Ki-67 positive cells **E.** and TUNEL positive cells **F.**. Values were mean ± SEM (*n* = 4). **P < 0.01, ***P < 0.001.

We have shown that HKI-272 exhibited superior inhibition of HER2 elicited signaling *in vitro* cells [14]. Studies from our transgenic mouse model showed that H878Y mutant HER2 function was important for the maintenance of tumor. To mimic clinical settings, we tested whether HKI-272 was effective in shrinking lung adenocarcinoma in transgenic mouse model. We fed a cohort of *CC10-rtTA/tet-op hHER2^H878Y^* mouse with Dox diet for one month and documented the tumor burden through CT imaging. Strikingly, we found that HKI-272 treatment for 2 weeks led to almost complete regression of the tumors, while tumors in placebo-treated group grew rapidly (Figure 4A,top two panels for CT images; Figure 4B for quantification). Histology data were consistent with imaging (Figure 4A, lower panel).

To see the effect of HKI-272 on HER2 downstream signals, we conducted immunoblotting analysis on the lung tissues. As expected, we found that HER2 downstream mediators, including PLCγ, STAT5, AKT and Cyclin D1, were decreased in tumor tissues lysates (Figure 4C). We further checked whether HKI-272 treatment led to apoptosis of tumor cells. As readouts for therapeutic efficacy, we evaluated proliferative (Ki-67) and apoptotic (TUNEL) marker *in situ* in the tumor cells by IHC. HKI-272 treatment for 3 days was effective in inhibiting tumor cell growth, as indicated by Ki-67 staining, and HKI-272 also eliciting tumor cell apoptosis, as indicated by TUNEL staining (Figure 4D for images; Figure 4E and 4F for quantification).

### Combinational treatment with HKI-272 and Rapamycin shows enhanced cytotoxicity to tumor driven by hHER2^H878Y^

Although HKI-272 is effective in shrinking H78Y mutant HER2 driven tumors, tumors in patients may be more complicated and combinational therapy may be necessary in those cases. The inhibitors preferably target on the same protein [23, 24], or distinct signaling components in the same pathway [25]. This was particularly relevant due to the release of feedback inhibition during targeting ErbB family members [26]. We therefore tested whether combinational therapy could enhance the cytotoxicity to tumors.

Of note, although we observed a reduction of AKT phosphoryation in HER2 transformed cells in both NIH-3T3 and BEAS-2B cellular background after treatment with HKI-272, residual phosphorylation of AKT signal was still to be detected (Figure 1A). AKT was an important effector for cell survival. Complete inhibition of AKT signaling in tumor cells would be ideal. Interestingly, the inhibition of the PI3K-Akt-mTOR signaling pathway with a PI3K and mTOR dual inhibitor (NVP-BEZ235) was shown to be effective in reducing viable HER2-driven cancer cells [27]. This suggests that Rapamycin might represent a suitable candidate for an adjuvant therapeutic modality that can be applied in combination with HKI-272 for treating H878Y mutant-driven cancers.

**Figure 5:**
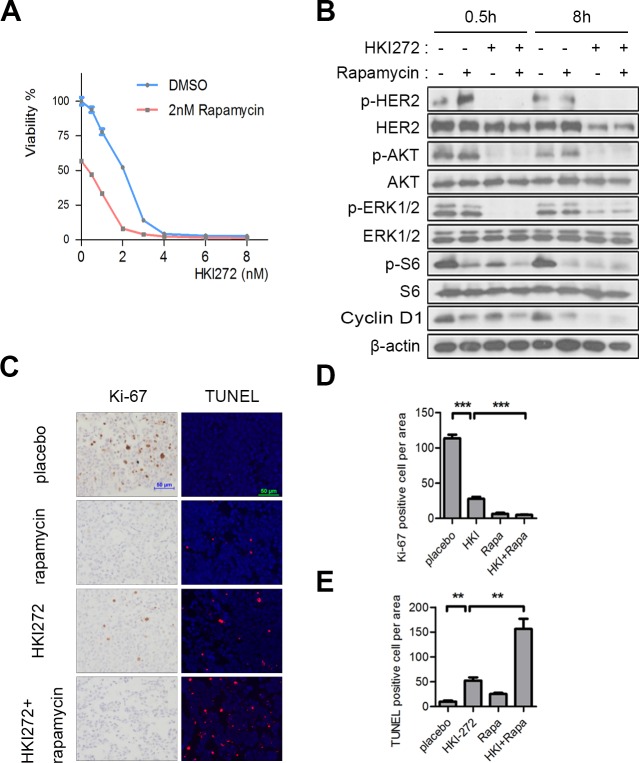
Rapamycin enhances the efficacy of HKI-272 towards HER2 H878Y-driven tumors **A.** H878Y transformed Ba/f3 cells were treated with HKI-272 alone or a combination of HKI-272 and 2nM Rapamycin for 3 days. Cell viability was determined by CellTiter-Glo assay. Values were mean ± SEM (*n* = 6). **B.** 3T3-H878Y cells were treated with DMSO, 500nM Rapamycin, 500nM HKI-272 or a combination of Rapamycin and HKI-272 for indicated time, and then subjected to immunoblotting analysis **C.**Representative photographs of cross-sectional tumors assayed for Ki-67 and TUNEL from treated mice. HER2 H878Y bitransgenic mouse were treated with vehicle, HKI-272, Rapamycin, or the combinational HKI-272/Rapamycin for 3 days respectively. Scale bars, 50μm. **D.**,**E.**Quantification of Ki-67 positive cells **D.** and TUNEL positive cells **E.** in the mouse **C.**. Values were mean ± SEM (*n* = 4). HKI, HKI-272; Rapa, Rapamycin. **P < 0.01,***P < 0.001.

We found that Rapamycin alone elicited a moderate growth inhibition in H878Y mutant transformed Ba/F3 cells, but not in WT HER2 transformed cells (Supplementary Figure 3), further suggesting that the increased AKT signaling elicited by the H878Y mutant rendered cells more sensitive to mTOR pathway inhibition. To further evaluate the potency of combinational treatment with HKI-272 and Rapamycin, we assessed the IC_50_ values of combined therapy with HKI-272 and Rapamycin compared to HKI-272 monotherapy. The IC_50_ of HKI-272 alone was approximately 2.4 nM and shifted a decrease to approximately 0.3 nM in the presence of 2 nM Rapamycin (Figure 5A). This finding showed that rapamycin synergized with HKI-272 to effectively kill H878Y HER2 mutant-transformed Ba/F3 cells. Consistently, we found that combinational treatment with Rapamycin and HKI-272 led to a decrease in signals of S6 phosphorylation and Cyclin D1 as well as inhibition of HER2, AKT and ERK phosphorylation in H878Y HER2 transformed 3T3 cells (Figure 5B). Our data showed that combined treatment with HKI-272 and rapamycin resulted in superior inhibition of signaling elicited by H878Y HER2 compared to either agent alone.

We further tested the combinational therapy *in vivo*. We treated a cohort of tumor-bearing bitransgenic mouse with vehicle, Rapamycin, HKI-272 and Rapamycin/HKI-272 combination. Rapamycin treatment for 3 days was effective in inhibiting tumor cell growth, as indicated by Ki-67 staining, but showned no evident effect in eliciting tumor cell death, as indicated by TUNEL staining (Figure 5C-5E). However, HKI-272 alone was effective both in tumor cell growth inhibition and apoptosis induction. Strikingly, we found that HKI-272/Rapamycin combinational therapy elicited significantly increased tumor cell death and inhibited cellular proliferation compared to either drug alone (Figure 5C-5E). These results showed that the combinational treatment was more toxic to tumor cells transformed by HER2 H878Y *in vivo*.

## DISCUSSION

Due to its high rate of deregulation (either overexpression or mutation) in various tumor types, HER2 is among the most intensely investigated drug targets. Mutations of HER2 within kinase domain are roughly grouped into two categories: activation loop and non-activation-loop mutations. H878Y is the only mutation located within activation loop reported for HER2. Our previous and present work have shown that H878Y HER2 is a gain-of-function mutation, which is tumorigenic *in vivo* in transgenic mouse model. More importantly, the tumor driven by this oncogene is dependent on its continuous expression for maintenance and these tumors can be effectively treated by a HER2 kinase inhibitor in this mouse model.

Activation loop of EGFR is frequently detected to be mutated in a variety of tumors. For example, L858R is one of the most potent mutations found in lung cancer patients [11]. These mutations are reported to enhance kinase activity of EGFR. Many researches have reported that these mutations are tumorigenic *in vivo* and that these mutations sensitize the tumor to EGFR TKI treatment. In contrast, H878Y is the only mutation reported in activation loop of HER2. We recently reported that H878Y was a gain-of-function mutation *in vitro* cell lines, and revealed a potential mechanism underlying this gain-of-function. Interestingly, we found H878Y mutation similar to L858R in EGFR in the way that it was a TKI-sensitizing mutation, which was different from 776 insertional mutation HER2^YVMA^. In our present work, we found that HKI-272, now entering clinic trial, was effective in treating H878Y mutant HER2 driven tumors. However, we had found that HKI-272 alone was not as effective as in H878Y case for treating tumors driven by HER2^YVMA^(data not shown).

Oncogenic HER2 was reported to activate some downstream effectors, such as PLCγ [28]. Considering the difference in locations within HER2 protein, the difference in the phenotypes of formed tumors, and the difference in drug sensitivity revealed in our work, HER2 H878Y and HER2^YVMA^ mutations may impact HER2 function in drastically different ways. Our data therefore highlighted the necessity and importance to further study the difference in signaling between H878Y and most other non-activatioin-loop-localizing mutations (such as HER2^YVMA^).

A number of HER2 inhibitory reagents are now available: small molecules and antibodies. Our earlier work reported that second generation inhitors such as BIBW-2992 and HKI-272 outperformed reversible inhibitors against HER2 transformed Ba/F3 cells in two ways: HKI-272 was less toxic to nontransformed cells, and HKI-272 was more potent in killing HER2 transformed cells. Antibodies can inhibit homo-dimerization of HER2 or hetero-dimerization with other members of ErbB family. Our work had reported that exon19 deletion mutant EGFR in lung cancers was not dependent on dimerization for functioning, and therefore, antibodies may be less effective on these mutations [29]. In the case of TKI resistance, combinational treatment with antibody and TKI is expected to kill HER2 transformed cells more effectively [30].

Of note, mTOR inhibition could upregulate the autophage. We noted in our mouse model that rapamycin synergized with HKI-272 to induce autophagy in H878Y driven lung tumors (data not shown).

Tumors in patients developed through long terms of inactivation of tumor suppressor genes and activation of growth promoting oncogenes, such that treatments that effectively killed tumor cell cell lines or shrank tumors in mouse models sometimes fail in patients. Combinational treatments based on reasonably designed schemes are preferable. We found that H878Y drove stronger activation of AKT signaling and residual AKT activation remained to be detected after HKI-272 treatment. We therefore tested Rapamycin, an inhibitor to block the mTOR signaling downstream of AKT to combine HKI-272 as a therapeutic strategy. We had confirmed the superior treatment effect in cell lines and in transgenic mouse models with this combined therapy, which caused tumor cell growth inhibition and apoptosis induction for HER2 H878Y mutant.

Our current work therefore revealed that H878Y mutant was tumorigenic *in vivo* and was a reasonable target for tumors driven by this HER2 mutation. Combinational treatment with HKI-272 and Rapamycin is effective *in vivo* mouse models, and warrant further validation in clinic.

## MATERIALS AND METHODS

### Mouse work

All mice were housed in a pathogen-free environment at the National Institute of Biological Sciences, Beijing (NIBS). All experimental protocols were approved by the Institutional Committee for Animal Care and Use at NIBS. All animal work was performed in accordance with the approved protocol.

### Cell lines

All cell lines used in this study were purchased from ATCC (American Type Culture Collection) and cultured in Dulbecco's Modified Eagle's Medium (DMEM) containing 10% FCS (for NIH-3T3, referred to as 3T3) or 10% FBS (for BEAS-2B). Ba/f3 cells were maintained in RPMI1640 medium containing 10%FBS and IL-3. Cell culture media were supplemented with 10 mM glutamine and 1% penicillin and streptomycin and incubated at 37°C in a 5% CO_2_ incubator.

### Sable cell line generation, Soft agar and cell viability assay

Plasmids constructs, stable cell lines generation, soft agar assay and cell viability assay were described as previous study [14].

### Western blotting

All cells or tissues were lysed in RIPA buffer (Beyotime) supplemented with protease and phosphatase inhibitors (Roche). Following lysis, the cells were sonicated. Western blotting was performed using standard methods. Antibody against β-actin was from Sigma, other antibodies were purchased from Cell Signaling Technology.

### Generation of the CC10-rtTA/tet-op-hHER2H878Y mouse cohort

To generate *Tet-op-hHER2^H878Y^* transgenic mice, human HER2 H878Y DNA was subcloned into pTRE-Tight (Clonetech), and then a β-globin intron was subcloned into this plasmid and just located between the Tet-responsive P_tight_ promoter and H878Y ORF. The DNA fragments containing *Tet-op-β-globin intron-hHER2 H878Y-SV40* polyA were injected into FVB/N blastocysts. The progeny were genotyped as described [26]. All mouse were housed in SPF room at NIBS' animal room and crossed with *CC10-rtTA* [26]. Genotyping of *CC10-rtTA* and *tet-op-hHER2^H878Y^* were performed by using following primers: for *CC10-rtTA*, forward primer: AAAATCTTGCCAGCTTTCCCC; reverse primer: ACTGCCCATTGCCCAAACAC. For *tet-op-hHER2^H878Y^*, forward primer: GGCACCCAGCTCTTTGAGGA; reverse primer: CACCTCTCGCAAGTGCTCCA.

### Drug treatment in vivo and micro-CT

All mice were housed in a pathogen-free environment at the National Institute of Biological Sciences, Beijing (NIBS). All mouse were handled strictly in accordance with good animal practice as defined by the Animal Facility in NIBS, and all animal work were done with approval from Institutional Committee for Animal Care and Use in NIBS. *CC10-rtTA/Tet-op-hHER2^H878Y^* mouse fed on Dox food when the pups reached 30 days old. After continuous exposure to doxycycline-containing diets (Research Diets) for 5 weeks, mouse were then randomized to groups and treated daily with 50 mg/kg HKI-272 (Selleck Chemicals) or 2mg/kg Rapamycin (Selleck Chemicals). HKI-272 was formulated in 0.5% methocellulose-0.4% Tween 80 and was administered by oral gavage, and Rapamycin formulated in 5%-PEG 400-5% Tween 80 was administrated by i.p. injection. Micro-CT analysis was performed using the Siemens Inveon PET-CT analyzer.

### H&E and IHC staining

Lungs were collected from animals and fixed overnight in 10% formalin supplied with 1mM NaF, 1mM NaVO3, 1mM PMSF. Hematoxylin and eosin (H&E) staining and Immuno-histological (IHC) staining were performed on 5μm formalin-fixed paraffin sections. The antibodies used were described in Western Blotting section. The ABC-Elite Rabbit IgG kit (Vector Laboratories) was used as secondary detection system (45min incubation at room temperature). DAB (Sigma) was used as a substrate. Ki-67 antibody (Vector Laboratories) was used to assess the proliferation of cells. Apoptosis was measured by TUNEL assay with TMR red In Situ Cell Death detection Kit (Roche) according to the manual. The nucleus was stained with DAPI.

## SUPPLEMENTARY MATERIAL FIGURES


