# Correction: Tumor driven by gain-of-function HER2 H878Y mutant is highly sensitive to HER2 inhibitor

**DOI:** 10.18632/oncotarget.26252

**Published:** 2018-10-16

**Authors:** Zexi Hu, Yong Hu, Xicheng Liu, Rongwen Xi, Aiqun Zhang, Deruo Liu, Qiang Xie, Liang Chen

**Affiliations:** ^1^ College of Life Sciences, Beijing Normal University, Beijing, China; ^2^ National Institute of Biological Sciences, Beijing, China; ^3^ The General Hospital of People’s Liberation Army (301 hospital), Beijing, China; ^4^ Department of thoracic surgery, China-Japan Friendship Hospital, Beijing, China; ^5^ Fuzhou Pulmonary Hospital of Fujian, Fujian, China; ^6^ National Institute of Biological Sciences, Collaborative Innovation Center for Cancer Medicine, Beijing, China

**This article has been corrected:** The correct Acknowledgment information is given below:

**ACKNOWLEDGMENTS**

This work was funded by National Natural Science Foundation of China grant 81472606, Chinese Ministry of Science and Technology grant (973 grant) 2011CB812401, and the Beijing Municipal Government.

Original article: Oncotarget. 2015; 6:31628-31639. https://doi.org/10.18632/oncotarget.5221

