# Supplementary material for: Tumor driven by gain-of-function HER2 H878Y mutant is highly sensitive to HER2 inhibitor
Source: Oncotarget. 2015 Sep 8;6(31):31628–39. doi: 10.18632/oncotarget.5221 (PMC4741629; doi:10.18632/oncotarget.5221)
Supplement: Supplementary file 1 [file oncotarget-06-31628-s001.pdf]

Tumor driven by gain-of-function HER2 H878Y mutant is highly sensitive to HER2 inhibitor

Supplementary Material

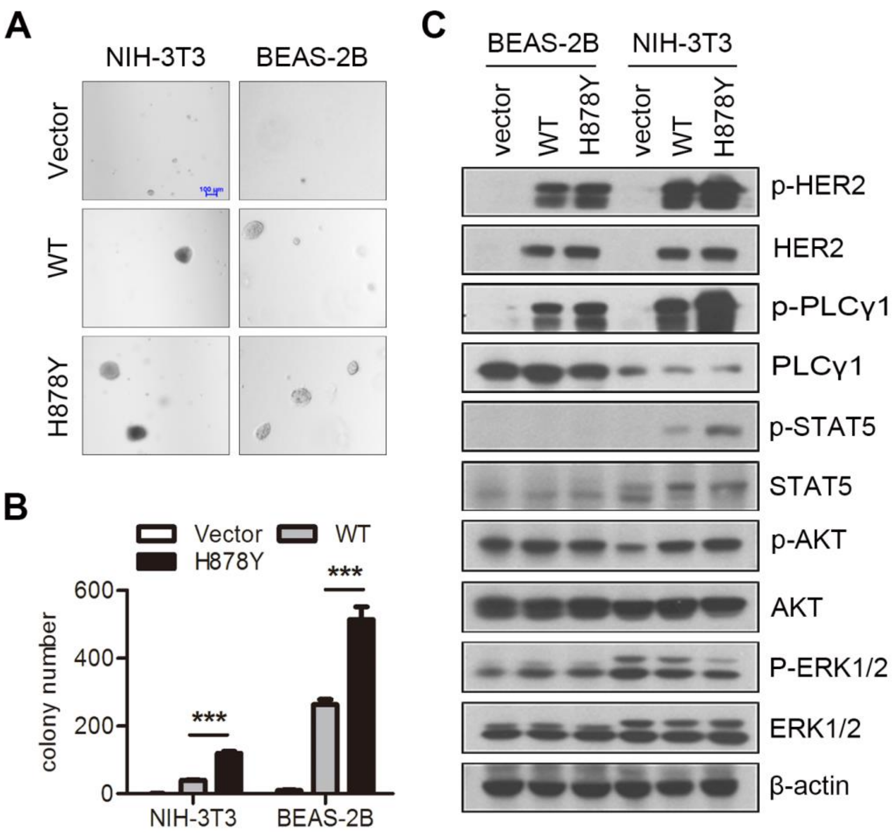

Supplementary Figure 1: HER2 H878Y is a gain-of-function mutation

(A), (B) Soft-agar assay on NIH-3T3 and BEAS-2B cells transfected with vector control, WT, and H878Y HER2 mutant (A) and quantification and statistics of the soft-agar results (B). Colonies > 100 µm were counted. Values were mean ± SEM (n=6). ****P*<0.001. H878Y transformed cells formed larger and more colonies on soft-agar assay. Scale bars, 100 µm.

(C) Immunoblotting analysis on HER2 expressing stable cell lines in NIH-3T3 and BEAS-2B showed active canonical downstream signals of HER2.

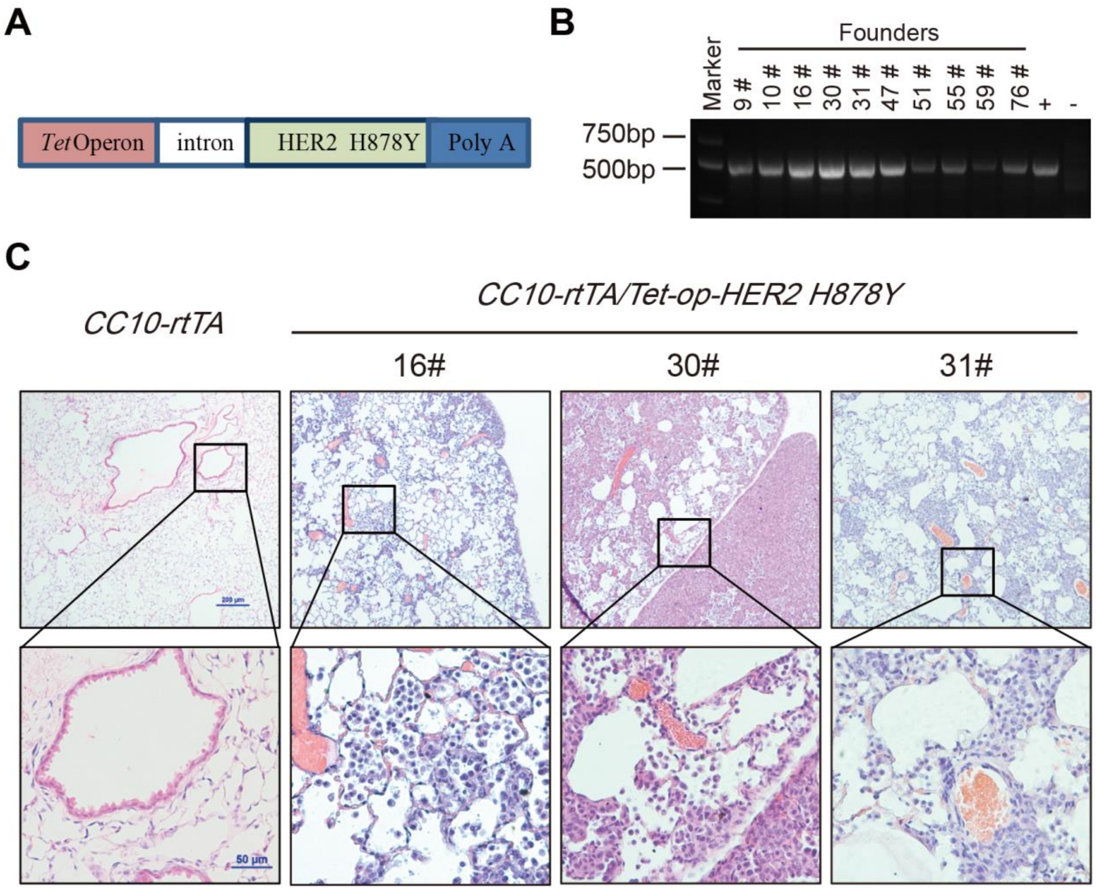

Supplementary Figure 2: Expression of H878Y induces mouse lung tumors

(A) Schematic diagram of Tet-op-HER2 H878Y transgene DNA fragment for transgenic mouse construction.

(B) Genotyping result of H878Y positive transgenic mouse founders.

(C) Lung-specific expression of H878Y induces lung adenocarcinoma with bronchioloalveolar carcinoma features. CC10-rtTA/tet-op-H878Y mouse cohorts from three different founder lines were fed with doxycycline food for 3 weeks, and then collected lung tissues for H&E staining. Scale bar, 200µm for upper panel, 50 µm for lower panel.

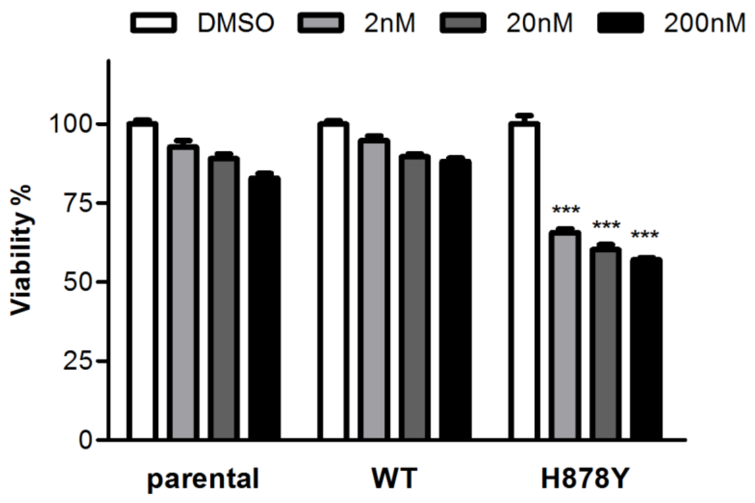

Supplementary figure 3: H878Y transformed Ba/f3 cells are more sensitive to Rapamycin than widetype HER2.

Parental or HER2 WT and H878Y transformed Ba/f3 cells were treated with Rapamycin at indicated concentration for 3 days. Cell viability was determined by CellTiter-Glo. Values were mean ± SEM (n= 7).
